# Integrative taxonomy of *Trichogramma vitessoides* sp. nov. (Hymenoptera, Trichogrammatidae), an egg parasitoid of *Heortia vitessoides* from southern China

**DOI:** 10.3897/zookeys.1295.211266

**Published:** 2026-10-14

**Authors:** Zhiqiang Ge, Longzhou Chen, Ziyao Xian, Tianyi Gu, Zhongqi Yang, Yanlong Zhang, Jiangshan Xin, Hongying Hu

**Affiliations:** 1 Xinjiang Key Laboratory of Biological Resources and Genetic Engineering, College of Life Science and Technology, Xinjiang University, Urumqi, China Xinjiang Key Laboratory of Biological Resources and Genetic Engineering, College of Life Science and Technology, Xinjiang University Urumqi China; 2 Forestry Institute of Maoming City, Maoming, China Forestry Institute of Maoming City Maoming China; 3 Ecology and Nature Conservation Institute, Chinese Academy of Forestry, Beijing, China Ecology and Nature Conservation Institute, Chinese Academy of Forestry Beijing China

**Keywords:** Biological control, DNA barcoding, male genitalia, mitochondrial genome, molecular phylogeny

## Abstract

Egg parasitoids of the genus *Trichogramma* Westwood, 1833 are widely used in integrated pest management, but their minute size and reduced morphology complicate identification. Using integrative taxonomy, we describe *Trichogramma vitessoides* Hu & Yang, **sp. nov**. from Guangdong, China, based on a laboratory colony established from parasitoids reared from naturally parasitised egg masses of *Heortia vitessoides* (Moore, 1885) (Lepidoptera, Crambidae), a defoliator of *Aquilaria sinensis* (Lour.) Spreng. Male genital morphology distinguishes the new species from morphologically similar congeners, particularly *Trichogramma raoi* Nagaraja, 1973, by differences in the shape of the dorsal extension, the aedeagus-to-apodeme ratio, and the length of the central ridges. The complete mitogenome is 16,221 bp in length, contains 37 genes, and has an A+T content of 84.4%. Bayesian and maximum-likelihood analyses based on the concatenated amino-acid sequences of the 13 mitochondrial protein-coding genes recovered the new species as sister to *Trichogramma japonicum* Ashmead, 1904. All 13 protein-coding genes had mean *Ka*/*Ks* ratios below 1, whereas *atp8* showed the highest ratio, suggesting comparatively relaxed purifying selection. These results support recognition of the new species and expand genomic resources for *Trichogramma* systematics and biological control.

## Introduction

Trichogrammatidae is a family of minute parasitoid wasps within the superfamily Chalcidoidea (Hymenoptera). Most trichogrammatids are egg parasitoids and have been recorded from more than 1,200 host species belonging to 11 insect orders and more than 90 families (Pinto 2006; Lin et al. 2022). Within the family, *Trichogramma* Westwood, 1833 is the most extensively studied genus and the most widely used in biological control programmes. More than 240 species of *Trichogramma* are currently recognised worldwide, including more than 30 species recorded from China (Lin et al. 2022; UCD Community 2023). Since the early twentieth century, *Trichogramma* species have been used as biological control agents against a wide range of insect pests. Their capacity to locate host eggs and suppress pest populations before the damaging larvae emerge has contributed to their widespread commercial production and field release (Smith 1996; Cônsoli et al. 2010). In China, mass-rearing and release programmes for *Trichogramma* have been developed for maize, rice, cotton, vegetable crops, and forest ecosystems, with releases covering tens of millions of hectares annually (Wang et al. 2014; Zang et al. 2021).

Despite their importance in biological control, accurate species identification within Trichogrammatidae remains challenging and may affect the selection of appropriate parasitoid species or strains for pest management. Adults are typically only 0.2–1.5 mm long, and several external characters vary with developmental temperature and host-related nutritional conditions, making closely related species difficult to distinguish (Nagarkatti and Nagaraja 1971). Species identification has therefore relied heavily on characters of the male genitalia, although these characters alone may be insufficient for resolving closely related species and cryptic lineages (Pinto 1999). Integrative taxonomic approaches combining detailed morphology with molecular markers, such as mitochondrial and nuclear sequences, are increasingly used to delimit and describe *Trichogramma* species (Takahashi et al. 2021).

DNA barcoding based on standardised gene regions, particularly mitochondrial *cox1*, provides a rapid means of comparing specimens and has become an important complement to morphology-based identification. However, a single short marker may contain insufficient phylogenetic information to distinguish recently diverged or closely related parasitoid species, and its performance may also be affected by incomplete reference databases (Hebert et al. 2004; Moritz and Cicero 2004; Smith et al. 2008). Complete mitochondrial genomes provide substantially more sequence characters than short barcode fragments and can therefore improve phylogenetic resolution among closely related taxa. Mitochondrial genomes are widely used in insect systematics because they are generally maternally inherited, occur in high copy numbers, and exhibit evolutionary rates suitable for comparisons at several taxonomic levels (Clayton 1982; Boore 1999). In addition to providing sequence data for phylogenetic reconstruction, complete mitogenomes allow comparisons of nucleotide composition, codon usage, and gene-order rearrangements (Cameron 2014; Zardoya 2020).

The present study arose from field surveys of insect pests and their natural enemies associated with *Aquilaria sinensis* (Lour.) Spreng., an economically and medicinally important tree cultivated in southern China. *A. sinensis* is an important source of agarwood and is damaged by the defoliating moth *Heortia vitessoides* (Moore, 1885), an economically important pest of *Aquilaria* species (Sajap 2013; Yan et al. 2020). Larvae of *H. vitessoides* feed gregariously within leaves bound with silk and, during severe infestations, may completely defoliate individual trees, reducing tree growth and potentially causing mortality (Sajap 2013; Yan et al. 2020). This sheltered feeding behaviour may reduce direct exposure to contact insecticides. Repeated insecticide applications against *H. vitessoides* in *A. sinensis* plantations have been reported to cause residue problems that reduce the quality and market value of agarwood (Qian et al. 2023). Identifying egg parasitoids associated with *H. vitessoides* may therefore provide candidates for future evaluation as biological control agents acting before the damaging larval stage.

In May 2024, we reared a morphologically distinctive *Trichogramma* species from naturally parasitised egg masses of *H. vitessoides* collected in an *A. sinensis* plantation in Dianbai District, Maoming, Guangdong Province. Previous laboratory experiments showed that the parasitoid can also complete development in eggs of the fall webworm, *Hyphantria cunea* (Drury, 1773) (Xin et al. 2025). Here, we assess the taxonomic status of this parasitoid using morphological examination, DNA barcode comparisons, complete mitochondrial genome characterisation, and phylogenetic analyses. We further compare relative selective constraints among the 13 mitochondrial protein-coding genes (PCGs) across the sampled *Trichogramma* species. The study provides an integrative taxonomic account of the new species, expands the available mitogenomic data for *Trichogramma*, and establishes a basis for future evaluation of its biological control potential.

## Materials and methods

### Insect sampling and laboratory colony establishment

Naturally parasitised egg masses of *Heortia vitessoides* were collected from the abaxial surfaces of *Aquilaria sinensis* leaves on 30 May 2024 at the Chunlong Kinam Agarwood Planting Professional Cooperative, Dianbai District, Maoming, Guangdong Province, China (21.68677°N, 111.35298°E). The egg masses were transferred to a controlled-environment chamber maintained at 25 °C, 70 ± 5% relative humidity, and a photoperiod of 16:8 h (L:D) until parasitoid emergence. After emergence, a laboratory colony was established and maintained on fresh eggs of *Hyphantria cunea* as a factitious host. Adult parasitoids were provided with a 10% honey solution. The holotype, paratypes, and all specimens used for morphological and molecular analyses were laboratory-reared descendants of the field-collected population after propagation on eggs of *Hyphantria cunea*. The holotype and paratypes are deposited in the Insect Collection of Xinjiang University, Urumqi, China (**ICXU**).

### Morphological examination

Six males, including the holotype, and six females were used for morphological examination and measurement. Specimens were cleaned, macerated, dehydrated, dissected, mounted in UV-curing resin on microscope slides, and dried before examination. Habitus images were captured using a Nikon SMZ25 stereomicroscope. Slide-mounted specimens were examined using a Nikon ECLIPSE Ci compound microscope, and detailed images were captured using a Nikon DS-U3 digital camera. Morphometric measurements were obtained from the digital images using ImageJ v. 1.8.0 (Schneider et al. 2012). Morphological terminology, abbreviations, and measurement definitions follow Pinto (1999). All measurements are given in millimetres. For genital measurements, ranges are followed by mean values in parentheses.

### DNA extraction and barcode amplification

Two laboratory-reared adult females were processed independently, and both molecular markers were amplified from each individual. Genomic DNA was extracted non-destructively using a TIANamp Genomic DNA Kit (Tiangen Biotech, Beijing, China) after incubation in lysis buffer for 12 h at 56 °C. The exoskeletons remaining after DNA extraction were retained as voucher specimens. Partial mitochondrial *cox1* and the D2 region of nuclear 28S rRNA were amplified using the primer pairs C1-J-1718/HCO2198 (Folmer et al. 1994; Simon et al. 1994) and D2-3551F/D2-4068R (Campbell et al. 1993), respectively. Each PCR was performed in a total volume of 25 μL containing 12.5 μL of 2× PreMix Taq (Tiangen Biotech, Beijing, China), 0.5 μL of each primer (10 μM), 5 μL of template DNA, and 6.5 μL of double-distilled water. The cycling profile for *cox1* consisted of an initial denaturation at 94 °C for 1 min; five cycles of 94 °C for 1 min, 45 °C for 1.5 min, and 72 °C for 1.5 min; 35 cycles of 94 °C for 1 min, 50 °C for 1.5 min, and 72 °C for 1 min; and a final extension at 72 °C for 5 min (Hebert et al. 2003). The cycling profile for 28S rRNA followed Campbell et al. (1993), with the annealing temperature adjusted to 55 °C: initial denaturation at 95 °C for 3 min; 30 cycles of 95 °C for 30 s, 55 °C for 30 s, and 72 °C for 30 s; and a final extension at 72 °C for 10 min. PCR products were visualised on 1% agarose gels and sequenced in both directions by Sangon Biotech (Shanghai, China).

### Sequence processing and genetic-distance analysis

For each specimen and molecular marker, forward and reverse chromatograms were assembled, trimmed to remove low-quality terminal regions, and manually checked in Geneious Prime v. 2025.1.2 to generate consensus sequences. Following trimming and sequence assembly, the two independently generated consensus sequences were identical in both length and nucleotide sequence for each marker. The *cox1* sequences (498 bp) were deposited in GenBank under accession numbers PZ626304 and PZ626305, while the 28S rRNA D2 sequences (577 bp) were deposited under PZ630113 and PZ630114. Because the two sequences were identical for each marker, one representative sequence per marker was used for the subsequent BLASTn searches, sequence alignment, and genetic-distance analysis. The representative sequences were compared against the GenBank non-redundant nucleotide database using BLASTn to identify similar sequences and obtain reference data for named *Trichogramma* species (Altschul et al. 1997). Reference and newly generated sequences were aligned using MAFFT v. 7 with the auto option and default parameters (Katoh and Standley 2013). Pairwise uncorrected p-distances and their standard errors were calculated for the *cox1* alignment in MEGA v. 12, with standard errors estimated from 1,000 bootstrap replicates (Kumar et al. 2024).

### High-throughput sequencing and data quality control

For mitogenome sequencing, 100 morphologically identified adult females from the laboratory colony maintained on eggs of *Hyphantria cunea* were pooled. Library preparation and high-throughput sequencing were performed by Sangon Biotech Co., Ltd. (Shanghai, China). A paired-end genomic library with an insert size of ~ 350 bp was constructed and sequenced on the Illumina NovaSeq X Plus platform using a PE150 strategy, generating ~ 6 GB of raw FASTQ data. Raw reads were quality-filtered using fastp v. 0.23.4 (Chen et al. 2018b) with the following parameters: -f 5 -t 5 -F 5 -T 5 -5 -3 -W 4 -M 20 -c -q 15 -u 40 -l 35. Only the resulting clean reads were used for mitogenome assembly.

### Mitogenome assembly, annotation, and visualisation

Initial *de novo* assembly and preliminary annotation of the mitochondrial genome were performed using MitoZ v. 3.6 (Meng et al. 2019) with the invertebrate mitochondrial genetic code and the following principal settings: --clade Arthropoda, --genetic_code 5, --fastq_read_length 150, --data_size_for_mt_assembly 0, --assembler megahit, --memory 50, and --requiring_taxa Arthropoda. MEGAHIT assemblies were generated using k-mer sizes of 71 and 99. As an independent comparison, a reference-guided assembly was generated in Geneious Prime v. 2025.1.2 by mapping the clean reads to the mitochondrial genome of *Trichogramma japonicum* Ashmead, 1904 (NC_039534). Concordance between the *de novo* and reference-guided assemblies was assessed by sequence alignment, and the final consensus sequence was manually inspected in Geneious Prime. Clean reads were subsequently mapped back to the final mitochondrial genome assembly in Geneious Prime, yielding an average sequencing depth of ~ 30× across the mitogenome. Gene boundaries were initially predicted using MITOS2 v. 2.1.9 (Bernt et al. 2013). Transfer RNA genes and their secondary structures were further examined using tRNAscan-SE v. 2.0 (Lowe and Chan 2016). All annotations were manually checked and adjusted in Geneious Prime by comparison with previously published *Trichogramma* mitogenomes. The circular mitochondrial genome map was generated using the Proksee web server (https://proksee.ca/; Grant et al. 2023).

### Mitogenomic sequence analyses

Nucleotide composition and strand asymmetry were calculated in MEGA v. 12. Strand asymmetry was expressed as AT-skew = (A – T) / (A + T) and GC-skew = (G – C) / (G + C) (Perna and Kocher 1995). Synonymous substitution rates (*Ks*), nonsynonymous substitution rates (*Ka*), and *Ka*/*Ks* ratios were calculated for orthologous PCGs across the eight sampled *Trichogramma* species using DnaSP v. 6.12.01 (Rozas et al. 2017). For each PCG, *Ka*/*Ks* ratios were calculated for all 28 pairwise comparisons among the eight sampled species, and the mean value across these comparisons was used to compare relative selective constraints among genes. Relative synonymous codon usage (RSCU) values for the 13 PCGs were calculated and visualised using PhyloSuite v. 1.2.3 (Zhang et al. 2020; Xiang et al. 2023). Interspecific synteny and potential gene-order rearrangements among the sampled *Trichogramma* mitogenomes were examined using the Mauve alignment algorithm (Darling et al. 2004) implemented in Geneious Prime.

### Amino-acid sequence alignment and phylogenetic reconstruction

Phylogenetic relationships were reconstructed using the amino-acid sequences of the 13 PCGs from eight *Trichogramma* species. *Pseudoligosita yasumatsui* (Viggiani & Subba Rao, 1978) (NC_082212) and *Megaphragma viggianii* Fusu, Polaszek & Polilov, 2022 (NC_028196, originally deposited as *M. amalphitanum* and subsequently reidentified as *M. viggianii* by Polaszek et al. 2022) were included as outgroups (Table 1). The amino-acid sequences of the 13 protein-coding genes were extracted and aligned separately using MAFFT v. 7 as implemented in PhyloSuite v. 1.2.3 (Katoh and Standley 2013; Zhang et al. 2020; Xiang et al. 2023). Poorly aligned regions were removed using Gblocks as implemented in PhyloSuite (Castresana 2000), and the trimmed amino-acid alignments were concatenated in PhyloSuite. Best-fitting amino-acid substitution models for Bayesian inference and maximum-likelihood analysis were selected using ModelFinder under the Bayesian information criterion (BIC) (Kalyaanamoorthy et al. 2017). The selected models were subsequently applied in the corresponding phylogenetic analyses. Bayesian inference (BI) was conducted in MrBayes v. 3.2.6 using two independent runs, each comprising four Markov chains, for 5,000,000 generations (Ronquist et al. 2012). Trees were sampled every 1,000 generations, and the first 25% of samples were discarded as burn-in. Convergence was assessed by confirming that the average standard deviation of split frequencies was below 0.01. Maximum-likelihood (ML) analysis was conducted in IQ-TREE v. 2.2.0 (Minh et al. 2020), with nodal support assessed using 5,000 ultrafast bootstrap replicates (Hoang et al. 2018). The resulting trees were visualised and annotated using tvBOT (Xie et al. 2023).

**Table 1.** Taxa and GenBank accession numbers used in the mitogenome-based phylogenetic analyses.

**No**.	**Family**	**Species**	**GenBank accession**	**Genome length (bp)**	**A+T content (%)**
1	Trichogrammatidae	*Trichogramma vitessoides* sp. nov.	PX395428	16,221	84.4
2	Trichogrammatidae	*Trichogramma pretiosum*	NC_069039	16,227	85.3
3	Trichogrammatidae	*Trichogramma ostriniae*	NC_039535	16,472	85.4
4	Trichogrammatidae	*Trichogramma kaykai*	PQ667799	16,399	85.2
5	Trichogrammatidae	*Trichogramma japonicum*	NC_039534	15,962	84.9
6	Trichogrammatidae	*Trichogramma dendrolimi*	KU836507	16,878	84.7
7	Trichogrammatidae	*Trichogramma chilonis*	MW789210	16,147	85.2
8	Trichogrammatidae	*Trichogramma cacaeciae*	NC_069040	16,034	84.9
9	Trichogrammatidae	*Pseudoligosita yasumatsui*	NC_082212	16,036	86.5
10	Trichogrammatidae	*Megaphragma viggianii*	NC_028196	15,041	85.3

Note: NC_028196 was originally deposited as *Megaphragma amalphitanum* but was subsequently reidentified as *M. viggianii* by Polaszek et al. (2022).

## Results

### Taxonomic account

#### Trichogramma vitessoides

Taxon classification

Animalia

HymenopteraTrichogrammatidae

Hu & Yang sp. nov.

5E2C9CF5-1A85-5B43-9628-50EDFE35E0EE

https://zoobank.org/099F43CA-E08F-4EC0-B8BC-117B6BA2EAD7

Figs 1, 2

##### Type material.

***Holotype***. China • ♂; Guangdong Province, Maoming, Dianbai District, Chunlong Kinam Agarwood Planting Professional Cooperative, 21.68677°N, 111.35298°E; laboratory-reared on eggs of *Hyphantria cunea* from a colony established from parasitoids emerging from naturally parasitised egg masses of *Heortia vitessoides* collected from *Aquilaria sinensis*; source population collected 30 May 2024; Xiang Wang leg.; slide-mounted in UV-curing resin; deposited in the Insect Collection of Xinjiang University, Urumqi, China (ICXU); ICXU-TRI-2024-001. ***Paratypes***. China • 5♂, 6♀; laboratory-reared descendants of the same colony as the holotype and maintained on eggs of *Hyphantria cunea*; slide-mounted in UV-curing resin; deposited in the Insect Collection of Xinjiang University, Urumqi, China (ICXU); ICXU-TRI-2024-002–012.

**Figure 1.**
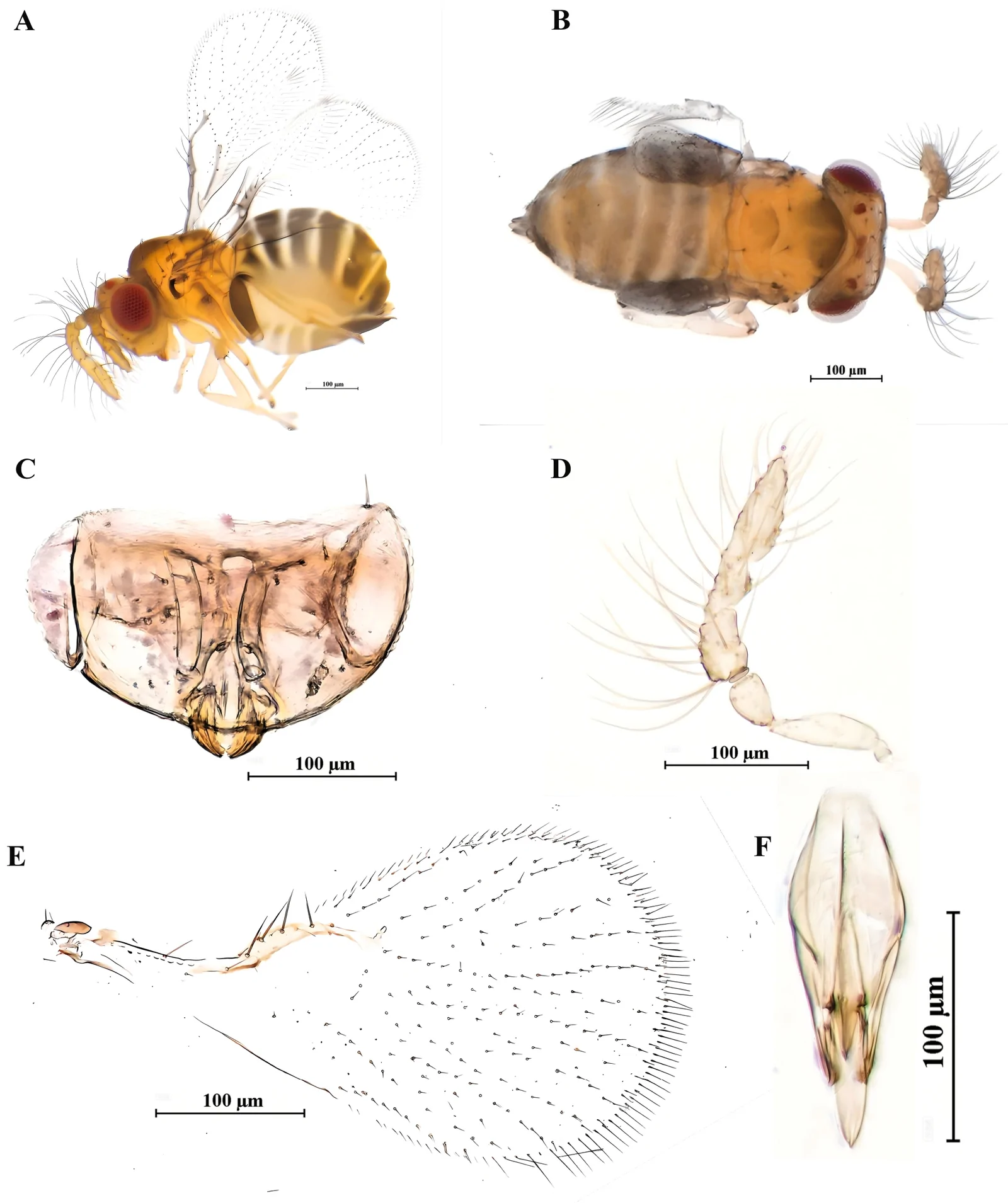
Male morphology of *Trichogramma vitessoides* sp. nov. **A**. Habitus, lateral view; **B**. Habitus, dorsal view; **C**. Head, frontal view; **D**. Antenna; **E**. Forewing; **F**. Male genital capsule.

**Figure 2.**
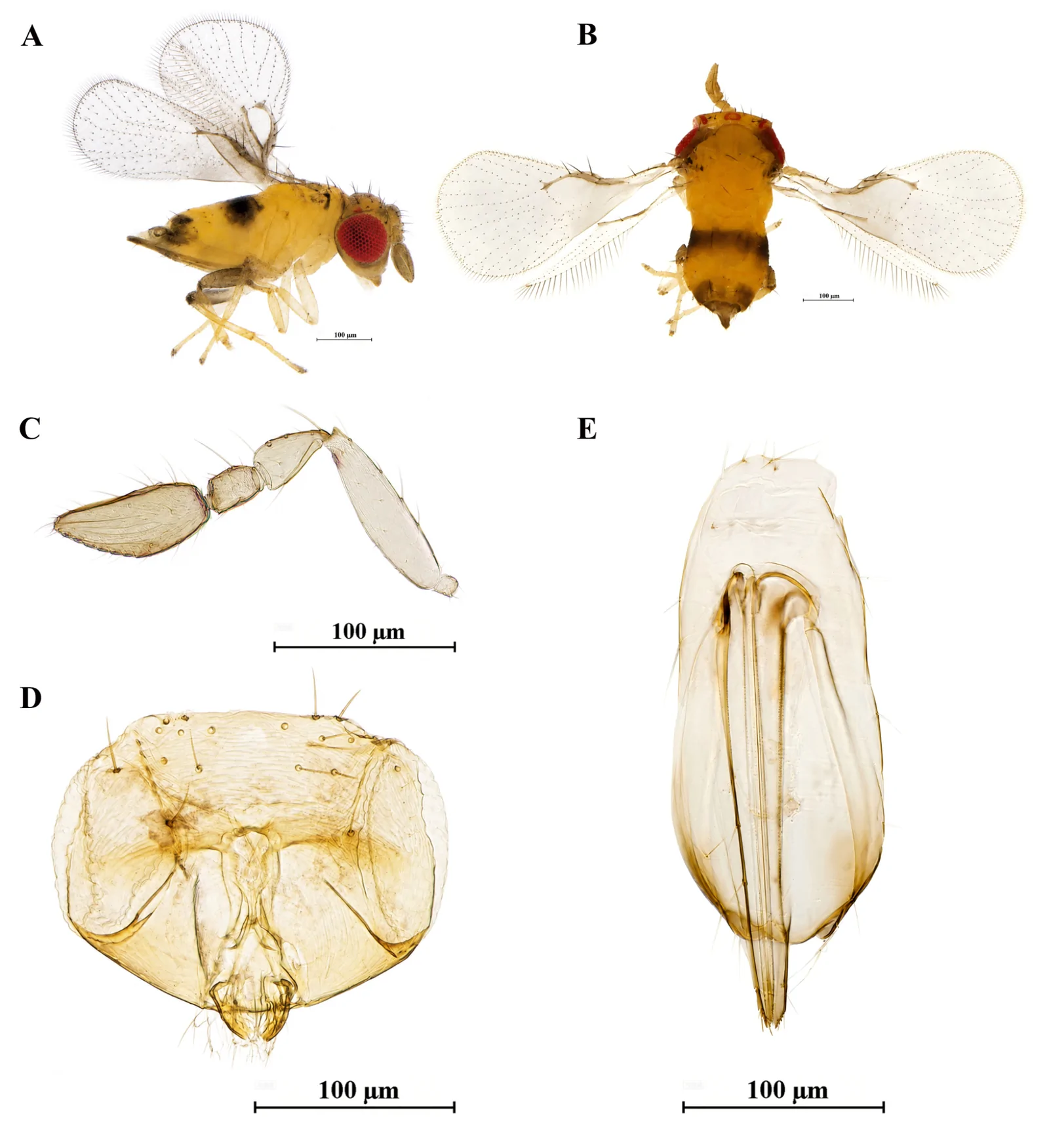
Female morphology of *Trichogramma vitessoides* sp. nov. **A**. Habitus, lateral view; **B**. Habitus, dorsal view; **C**. Antenna; **D**. Head, frontal view; **E**. Ovipositor.

##### Additional non-type material.

China • one ethanol-preserved colony lot consisting of additional laboratory-reared specimens from the same colony and maintained under the same rearing conditions as the type series; preserved in 99% ethanol; deposited in the Insect Collection of Xinjiang University, Urumqi, China (ICXU); ICXU-TRI-2024-013.

##### Diagnosis.

*Trichogramma vitessoides* sp. nov. can be distinguished from morphologically similar congeners by the following combination of male genital characters: dorsal extension of the genital capsule without lateral lobes; chelate structures straight, with their apices slightly exceeding or approximately level with the apices of the gonoforceps; median ventral projection weakly developed; central ridges long, extending 0.85–0.93× the basal distance; aedeagus distinctly longer than its apodemes, with an aedeagus-to-apodeme ratio of 1.15–1.42; and the combined length of the aedeagus and apodemes is 0.55–0.68 × the hind tibial length. The new species is morphologically most similar to *Trichogramma raoi* Nagaraja, 1973, but differs in the relative positions of the apices of the chelate structures and gonoforceps and in having the aedeagus distinctly longer than its apodemes.

**Male (*n* = 6, including the holotype)**. Body length 0.394–0.571 mm; head width 0.202–0.262 mm; hind tibial length 0.157–0.178 mm (Fig. 1A–C). General body colour yellow, with dark brown markings on the lateral regions of the pronotum, median region of the mesoscutum, hind femora, and metasomal tergites. Antennal flagellum 4.48–6.57× as long as wide; longest flagellar seta 3.1–3.5× the maximum width of the flagellum (Fig. 1D). Forewing broad and hyaline, ~ 1.9× as long as wide; Rs1 track distinct and bearing three setae (Fig. 1E). Male genital capsule with dorsal extension (DEG) lacking lateral lobes. Chelate structures (CS) straight, 0.034–0.040 mm long (mean 0.036 mm). Gonoforceps (GF) 0.032–0.036 mm long (mean 0.034 mm), slightly shorter than or equal in length to the CS (GF/CS = 0.89–1.00, mean 0.94); apex of GF slightly proximal to or approximately level with apex of CS. Median ventral projection (MVP) weakly developed, 0.018–0.026 mm long (mean 0.022 mm). Central ridges (CR) prominent and long, 0.088–0.098 mm (mean 0.093 mm), extending nearly to the basal margin of the genital capsule and measuring 0.85–0.93× the basal distance (BD = 0.102–0.112 mm, mean 0.107 mm). Aedeagus (ae) 0.048–0.068 mm long (mean 0.060 mm), distinctly longer than its apodemes (ap), which measure 0.038–0.052 mm (mean 0.047 mm); ae/ap = 1.15–1.42 (mean 1.28). Combined length of the aedeagus and apodemes (ADG) 0.086–0.116 mm (mean 0.108 mm), equivalent to 0.55–0.68× the hind tibial length (mean 0.65) and 0.63–0.81× the genital capsule length (mean 0.76) (Fig. 1F).

**Female (*n* = 6)**. Body length 0.419–0.560 mm; head width 0.208–0.252 mm; hind tibial length 0.125–0.172 mm (Fig. 2A, B, D). General body colour yellow, with brown markings on the hind femora and basal and apical metasomal tergites; mesosoma otherwise yellow. Antennal clava 0.85–1.15× as long as the scape (Fig. 2C). Ovipositor 1.51–1.73× as long as the hind tibia (Fig. 2E).

##### Natural host.

*Heortia vitessoides* (Moore, 1885) (Lepidoptera, Crambidae).

##### Factitious host.

*Hyphantria cunea* (Drury, 1773) (Lepidoptera, Erebidae), used for laboratory colony maintenance.

##### Distribution.

Known only from the type locality in Maoming, Guangdong Province, China.

##### Etymology.

The specific epithet refers to the natural lepidopteran host, *Heortia vitessoides*, from which the original parasitoid colony was obtained.

##### Molecular data.

GenBank accessions: complete mitochondrial genome, PX395428; partial *cox1* sequences, PZ626304 and PZ626305; 28S rRNA D2 sequences, PZ630113 and PZ630114.

##### Remarks.

*Trichogramma vitessoides* Hu & Yang, sp. nov. differs from *Trichogramma pretiosum* Riley, 1879 in having more extensively developed chelate structures and central ridges; in *T. pretiosum*, the chelate structures extend to approximately half the length of the dorsal extension, the median ventral projection is minute, and the central ridges are paired and shorter than half the basal distance. In *Trichogramma fuentesi* Torre, 1980, the chelate structures likewise reach only approximately half the dorsal extension, the median ventral projection is level with the chelate structures, the central ridges extend to ~ 0.3× the basal distance, and the gonoforceps are moderately swollen. In *Trichogramma minutum* Riley, 1871 and *Trichogramma platneri* Nagarkatti, 1975, the chelate structures also extend to approximately half the dorsal extension; the median ventral projection does not reach the chelate structures in *T. minutum* but nearly reaches them in *T. platneri*. In *Trichogramma evanescens* Westwood, 1833, the median ventral projection is prominent and sharply triangular, the central ridges extend 0.25–0.43× the basal distance, and the combined length of the aedeagus and apodemes is slightly greater than the genital capsule length. The new species is most similar to *T. raoi*, but in that species the apex of the chelate structures lies slightly proximal to the apex of the gonoforceps and the aedeagus is approximately equal in length to its apodemes. In *T. vitessoides* sp. nov., the chelate structures are level with or slightly exceed the gonoforceps, and the aedeagus is distinctly longer than its apodemes (Pinto 1999; Lin et al. 2022). The name “*Trichogramma vitessoides*” was used informally in an earlier biological study by Xin et al. (2025), but the species is formally described and its name-bearing types are designated here.

### DNA barcode comparisons

Bidirectional sequencing of *cox1* and the 28S rRNA D2 region from two independently processed females yielded identical consensus sequences for each marker. The two *cox1* sequences were 498 bp long and were deposited in GenBank under accession numbers PZ626304 and PZ626305, whereas the two 28S rRNA D2 sequences were 577 bp long and were deposited under accession numbers PZ630113 and PZ630114. Comparison with the corresponding region of the complete mitochondrial genome (PX395428) showed near-complete concordance, with only a few discrepant bases at the ends of the bidirectional Sanger reads. BLASTn searches of the representative *cox1* sequence recovered the highest identities with two unidentified *Trichogramma* sequences, OP443545 and OP443462, which showed 99.37% and 99.16% identity, respectively, > 95% query coverage (Table 2). Among the named *Trichogramma* sequences included in the top BLAST hits, sequence identity ranged from 92.17% to 92.68%. Identity was 92.57% with *T. japonicum* (MF624062 and NC_039534), 92.68% with *Trichogramma chilonis* Ishii, 1941 (MT712144 and MW789210), and 92.17% with *Trichogramma cacaeciae* Marchal, 1927 (NC_069040). Uncorrected *cox1* p-distances between *T. vitessoides* sp. nov. and named congeners ranged from 0.0726 to 0.0812 (Table 3). The lowest distance was observed with *Trichogramma kaykai* Pinto & Stouthamer, 1997 (0.0726), followed by *T. chilonis* (0.0748), whereas the distance to the mitogenome-inferred sister species *T. japonicum* was 0.0769 (SE = 0.0128). BLASTn analysis of the 28S rRNA D2 sequence recovered a maximum identity of 94.42% with *Trichogramma mwanzai* Schulten & Feijen, 1982 (MT199196), followed by 94.34% with *T. pretiosum* (AY599408) and *T. minutum* (AY623514), 94.00% with *T. platneri* (AY599407), and 93.83% with *T. fuentesi* (AY599406) (Table 4).

**Table 2.** Top BLASTn matches for the partial *cox1* sequence of *Trichogramma vitessoides* sp. nov.

**Scientific name**	**Max score**	**Query coverage (%)**	**E-value**	**Identity (%)**	**Sequence length (bp)**	**Accession number**
*Trichogramma* sp.	859	95	0	99.37	652	OP443545
*Trichogramma* sp.	854	95	0	99.16	652	OP443462
*Trichogramma japonicum*	715	100	0	92.57	518	MF624062
*Trichogramma japonicum*	715	100	0	92.57	15,962	NC_039534
*Trichogramma kaykai*	715	100	0	92.57	16,399	PQ667799
*Trichogramma chilonis*	710	99	0	92.68	16,176	MT712144
*Trichogramma chilonis*	710	99	0	92.68	16,147	MW789210
*Trichogramma bilingensis*	704	100	0	92.17	518	MF624048
*Trichogramma mwanzai*	704	100	0	92.17	518	DQ177920
*Trichogramma cacaeciae*	704	100	0	92.17	16,034	NC_069040

**Table 3.** Uncorrected p-distances among *Trichogramma vitessoides* sp. nov. and selected *Trichogramma* sequences based on partial *cox1*.

**Species**	**1**	**2**	**3**	**4**	**5**	**6**	**7**	**8**	**9**	**10**	**11**
1 *Trichogramma vitessoides* sp. nov.		0.0037	0.0037	0.0128	0.0128	0.0127	0.0121	0.0121	0.0125	0.0125	0.0129
2 *Trichogramma* sp. OP443545	0.0064		0.0000	0.0122	0.0122	0.0123	0.0123	0.0123	0.0131	0.0121	0.0130
3 *Trichogramma* sp. OP443462	0.0064	0.0000		0.0122	0.0122	0.0123	0.0123	0.0123	0.0131	0.0121	0.0130
4 *Trichogramma japonicum*MF624062	0.0769	0.0705	0.0705		0.0000	0.0122	0.0129	0.0129	0.0136	0.0129	0.0128
5 *Trichogramma japonicum*NC_039534	0.0769	0.0705	0.0705	0.0000		0.0122	0.0129	0.0129	0.0136	0.0129	0.0128
6 *Trichogramma kaykai*PQ667799	0.0726	0.0684	0.0684	0.0726	0.0726		0.0119	0.0119	0.0123	0.0119	0.0113
7 *Trichogramma chilonis*MT712144	0.0748	0.0769	0.0769	0.0812	0.0812	0.0684		0.0000	0.0090	0.0117	0.0119
8 *Trichogramma chilonis*MW789210	0.0748	0.0769	0.0769	0.0812	0.0812	0.0684	0.0000		0.0090	0.0117	0.0119
9 *Trichogramma bilingensis*MF624048	0.0791	0.0855	0.0855	0.0876	0.0876	0.0726	0.0385	0.0385		0.0116	0.0124
10 *Trichogramma mwanzai*DQ177920	0.0791	0.0748	0.0748	0.0812	0.0812	0.0705	0.0641	0.0641	0.0641		0.0127
11 *Trichogramma cacaeciae*NC_069040	0.0812	0.0812	0.0812	0.0769	0.0769	0.0662	0.0684	0.0684	0.0726	0.0791	

Note: Pairwise uncorrected p-distances are shown below the diagonal, and bootstrap standard errors based on 1,000 replicates are shown above the diagonal. The distance between *Trichogramma vitessoides* sp. nov. and its mitogenome-inferred sister taxon, *T. japonicum*, was 0.0769, whereas distances to the sampled named congeners ranged from 0.0726 to 0.0812.

**Table 4.** Top BLASTn matches for the 28S rRNA D2 sequence of *Trichogramma vitessoides* sp. nov.

**Scientific name**	**Max score**	**Total score**	**Query coverage (%)**	**E-value**	**Identity (%)**	**Sequence length (bp)**	**Accession number**
*Trichogramma pretiosum*	885	885	100	0	94.34	963	AY599408
*Trichogramma minutum*	885	885	100	0	94.34	964	AY623514
*Trichogramma mwanzai*	874	874	99	0	94.42	593	MT199196
*Trichogramma platneri*	874	874	100	0	94.00	963	AY599407
*Trichogramma mwanzai*	870	870	99	0	94.24	593	MT199195
*Trichogramma fuentesi*	869	869	100	0	93.83	964	AY599406
*Trichogramma parkeri*	839	839	100	0	92.93	962	AY623513
*Trichogrammatoidea* sp.	793	793	100	0	91.54	958	AY623511
*Trichogramma funiculatum*	785	785	100	0	91.38	954	AY623512
*Trichogramma* sp.	721	721	84	0	93.47	511	KY934081

### Mitogenome organisation and nucleotide composition

**Figure 3.**
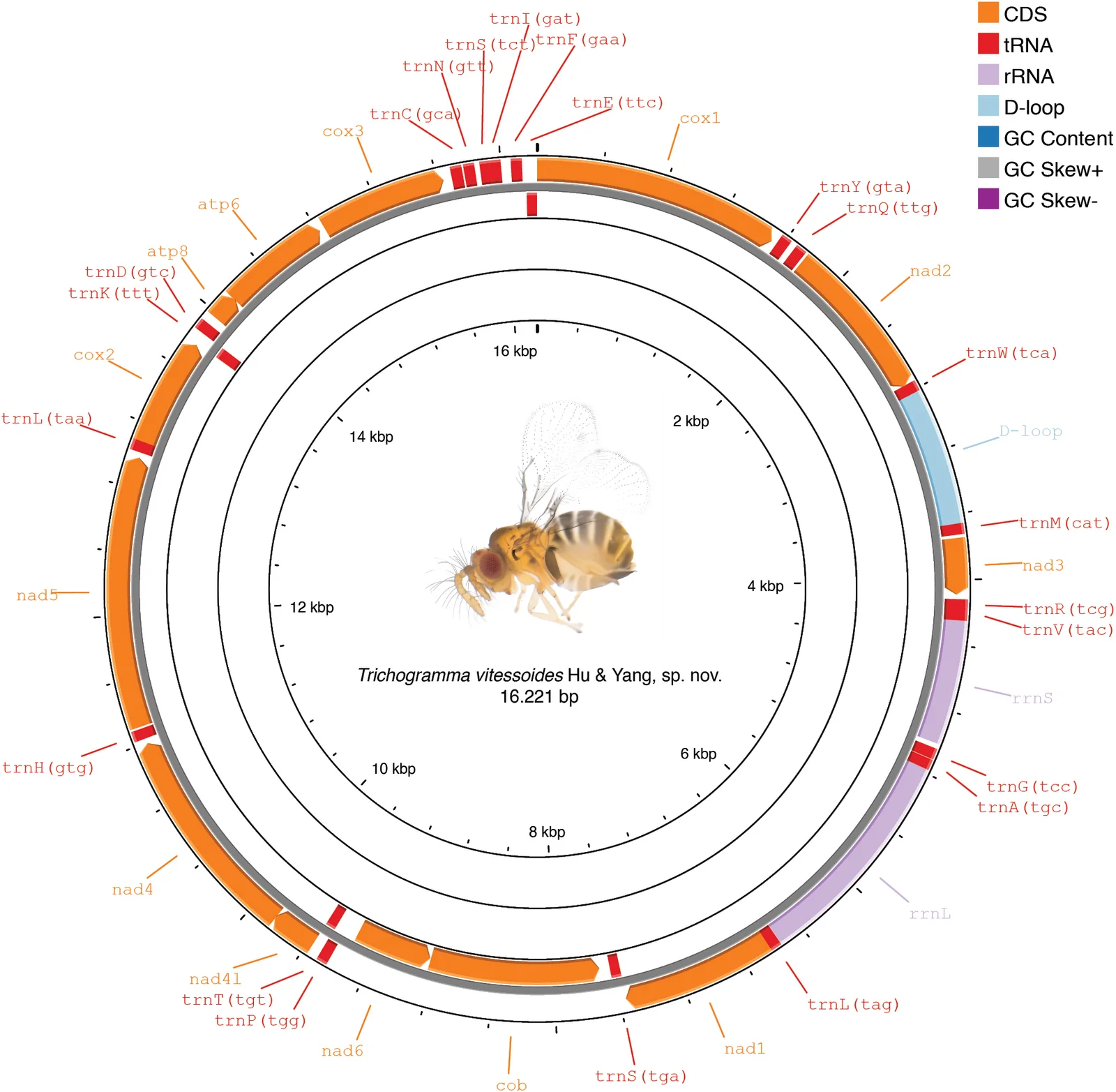
Circular map of the mitochondrial genome of *Trichogramma vitessoides* sp. nov. Gene names are shown using standard mitochondrial abbreviations. The inner circles indicate GC content and GC skew.

**Figure 4.**
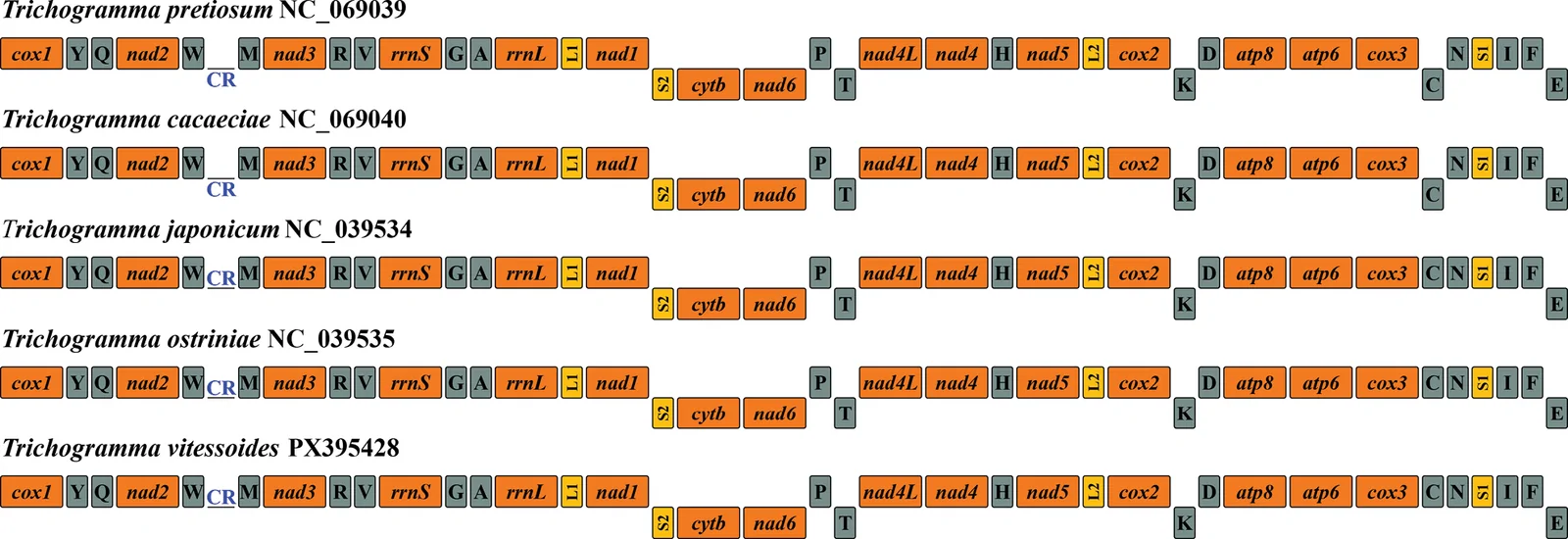
Mauve alignment of mitochondrial genomes from *Trichogramma vitessoides* sp. nov. and selected congeners.

The mitochondrial genome of *T. vitessoides* sp. nov. is 16,221 bp long and contains the typical set of 37 mitochondrial genes, comprising 13 PCGs, 22 tRNA genes, and two rRNA genes, together with one non-coding control region (D-loop) (Figs 3, 4). Among the sampled *Trichogramma* mitogenomes included in the comparison, the relative order of the 37 mitochondrial genes was conserved, although the strand orientation of some genes differed among species. According to the final annotation, the H strand encodes 31 genes, including 11 PCGs, 18 tRNAs, and both rRNAs, whereas the L strand encodes six genes, namely *cob*, *nad6*, *trnS2*, *trnT*, *trnK*, and *trnE* (Table 5). Seven gene overlaps were identified, the longest being a 7-bp overlap between *nad4L* and *nad4*. The genome also contained 25 intergenic spacers, with the longest spanning 86 bp between *nad6* and *trnP*. The complete mitogenome had an A+T content of 84.4%, an AT-skew of –0.122, and a GC-skew of 0.190. The negative AT-skew indicates a higher frequency of T than A, whereas the positive GC-skew indicates a higher frequency of G than C. All major genomic regions were A+T-rich, with the D-loop showing the highest A+T content of 88.9% (Table 6).

**Table 5.** Mitochondrial genome organization of *Trichogramma vitessoides* sp. nov.

**Gene**	**From**	**To**	**Length (bp)**	**Intergenic nucleotides (bp)**	**Anticodon**	**Start codon**	**Stop codon**	**Strand**
*cox1*	1	1,530	1,530	2	-	ATG	TAA	H
*trnY*	1,566	1,631	66	35	GTA	-	-	H
*trnQ*	1,673	1,740	68	41	TTG	-	-	H
*nad2*	1,759	2,754	996	18	-	ATT	TAA	H
*trnW*	2,753	2,817	65	-2	TCA	-	-	H
D-loop	2,818	3,657	840	0	-	-	-	H
*trnM*	3,658	3,724	67	0	CAT	-	-	H
*nad3*	3,746	4,090	345	21	-	ATA	TAA	H
*trnR*	4,121	4,183	63	30	TCG	-	-	H
*trnV*	4,182	4,248	67	-2	TAC	-	-	H
*rrnS*	4,249	5,008	760	0	-	-	-	H
*trnG*	5,041	5,106	66	32	TCC	-	-	H
*trnA*	5,112	5,175	64	5	TGC	-	-	H
*rrnL*	5,176	6,554	1,379	0	-	-	-	H
*trnL1*	6,559	6,625	67	4	TAG	-	-	H
*nad1*	6,623	7,561	939	-3	-	ATA	TAA	H
*trnS2*	7,560	7,623	64	-2	TGA	-	-	L
*cob*	7,695	8,834	1,140	71	-	ATG	TAA	L
*nad6*	8,842	9,348	507	7	-	ATT	TAA	L
*trnP*	9,435	9,500	66	86	TGG	-	-	H
*trnT*	9,500	9,563	64	-1	TGT	-	-	L
*nad4L*	9,568	9,855	288	4	-	ATT	TAG	H
*nad4*	9,849	11,192	1,344	-7	-	ATG	TAA	H
*trnH*	11,227	11,290	64	34	GTG	-	-	H
*nad5*	11,305	12,987	1,683	14	-	ATT	TAA	H
*trnL2*	13,022	13,087	66	34	TAA	-	-	H
*cox2*	13,088	13,768	681	0	-	ATT	TAA	H
*trnK*	13,781	13,850	70	12	TTT	-	-	L
*trnD*	13,852	13,917	66	1	GTC	-	-	H
*atp8*	13,975	14,142	168	57	-	ATT	TAA	H
*atp6*	14,136	14,810	675	-7	-	ATG	TAA	H
*cox3*	14,847	15,638	792	36	-	ATG	TAG	H
*trnC*	15,693	15,761	69	54	GCA	-	-	H
*trnN*	15,774	15,839	66	12	GTT	-	-	H
*trnS1*	15,873	15,932	60	33	TCT	-	-	H
*trnI*	15,933	15,999	67	0	GAT	-	-	H
*trnF*	16,062	16,127	66	62	GAA	-	-	H
*trnE*	16,153	16,219	67	25	TTC	-	-	L

Note: Positive values indicate intergenic spacers, whereas negative values indicate gene overlaps. H and L denote the two annotated mitochondrial strands, respectively.

**Table 6.** Nucleotide composition and strand asymmetry of the mitochondrial genome of *Trichogramma vitessoides* sp. nov.

**Regions**	**Length (bp)**	**T (%)**	**C (%)**	**A (%)**	**G (%)**	**A+T (%)**	**G+C (%)**	**AT-skew**	**GC-skew**
PCGs	11,088	48.6	7.6	33.4	10.3	82.0	17.9	-0.186	0.149
*atp6*	675	47.9	9.6	32.6	9.9	80.5	19.5	-0.190	0.015
*atp8*	168	54.2	6.0	33.9	6.0	88.1	12.0	-0.230	0.000
*cox1*	1,530	45.5	10.7	30.5	13.3	76.0	24.0	-0.198	0.109
*cox2*	681	44.6	7.8	35.1	12.5	79.7	20.3	-0.120	0.232
*cox3*	792	48.9	8.2	29.9	13.0	78.8	21.2	-0.240	0.226
*cob*	1,140	41.1	12.0	36.1	10.9	77.2	22.9	-0.065	-0.050
*nad1*	939	49.6	6.2	32.7	11.5	82.3	17.7	-0.206	0.301
*nad2*	996	52.1	4.9	36.8	6.1	88.9	11.0	-0.172	0.109
*nad3*	345	49.6	5.5	34.5	10.4	84.1	15.9	-0.179	0.309
*nad4*	1,344	52.1	6.6	31.4	9.9	83.5	16.5	-0.248	0.198
*nad4L*	288	58.7	2.4	29.9	9.0	88.6	11.4	-0.325	0.576
*nad5*	1,683	51.7	5.7	32.8	9.8	84.5	15.5	-0.224	0.264
*nad6*	507	45.2	7.1	43.0	4.7	88.2	11.8	-0.025	-0.200
tRNAs	1,448	45.0	4.3	43.2	7.5	88.2	11.8	-0.021	0.275
rRNAs	2,139	44.8	4.4	43.5	7.2	88.3	11.6	-0.015	0.240
*rrnL*	1,379	44.3	4.6	44.0	7.0	88.3	11.6	-0.003	0.205
*rrnS*	760	45.8	4.1	42.5	7.6	88.3	11.7	-0.037	0.303
D-loop	840	43.7	4.5	45.2	6.5	88.9	11.1	0.017	0.183
Full genome	16,221	47.3	6.3	37.1	9.3	84.4	15.6	-0.122	0.190

### Protein-coding genes and codon usage

**Figure 5.**
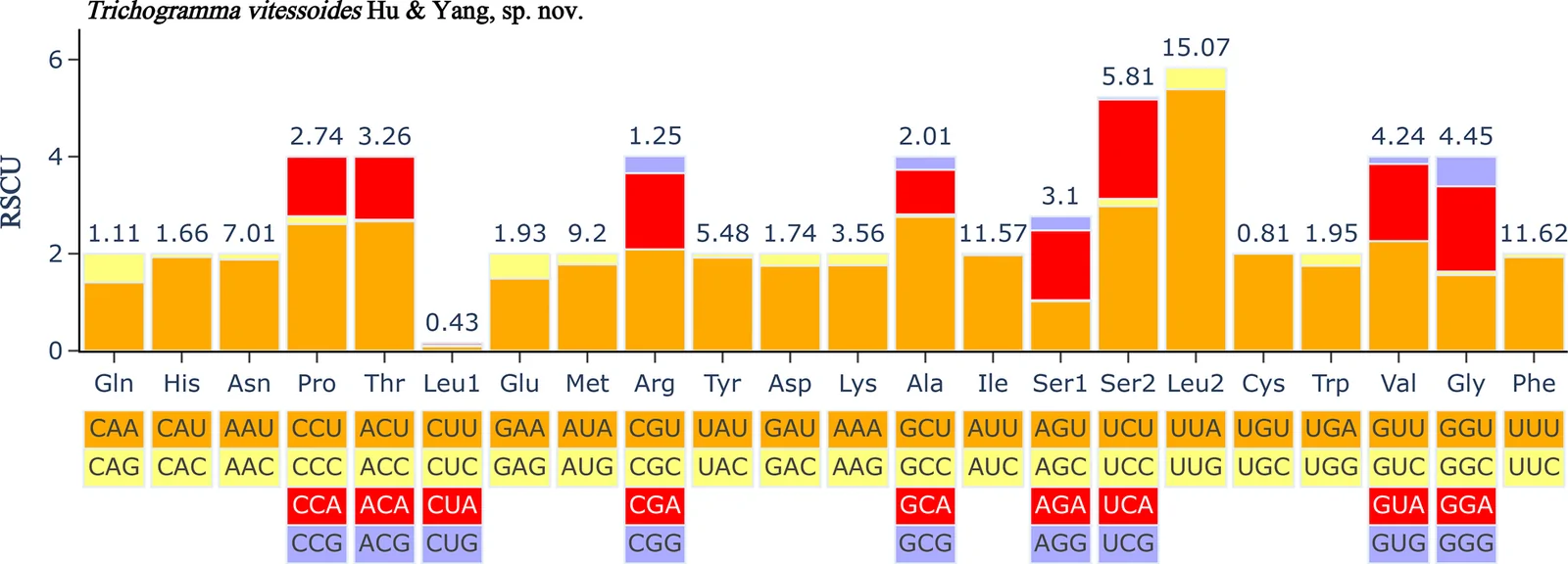
Relative synonymous codon usage of the 13 mitochondrial protein-coding genes of *Trichogramma vitessoides* sp. nov.

The 13 PCGs have a combined length of 11,088 bp, accounting for 68.4% of the complete mitochondrial genome. *nad5* is the longest PCG at 1,683 bp, whereas *atp8* is the shortest at 168 bp. The combined A+T content of the PCGs is 82.0% (Table 6). All PCGs begin with ATN start codons. *cox1*, *cob*, *nad4*, *atp6*, and *cox3* use ATG; *nad2*, *nad6*, *nad4L*, *nad5*, *cox2*, and *atp8* use ATT; and *nad3* and *nad1* use ATA. Eleven PCGs terminate with TAA, whereas *nad4L* and *cox3* terminate with TAG (Table 5). Codon usage was biased towards codons ending in A or U, consistent with the high A+T content of the genome. Leucine, phenylalanine, and isoleucine were the most frequently encoded amino acids, whereas cysteine was the least frequent (Table 7; Fig. 5).

**Table 7.** Codon usage and relative synonymous codon usage values for the 13 mitochondrial protein-coding genes of *Trichogramma vitessoides* sp. nov.

**Codon**	**Count**	**RSCU**	**Codon**	**Count**	**RSCU**	**Codon**	**Count**	**RSCU**	**Codon**	**Count**	**RSCU**
UUU (F)	412	1.93	UCU (S)	122	2.98	UAU (Y)	194	1.92	UGU (C)	30	2.00
UUC (F)	16	0.07	UCC (S)	6	0.15	UAC (Y)	8	0.08	UGC (C)	0	0.00
UUA (L)	513	5.39	UCA (S)	84	2.05	UAA (*)	11	1.69	UGA (W)	63	1.75
UUG (L)	42	0.44	UCG (S)	2	0.05	UAG (*)	2	0.31	UGG (W)	9	0.25
CUU (L)	9	0.09	CCU (P)	66	2.61	CAU (H)	59	1.93	CGU (R)	24	2.09
CUC (L)	2	0.02	CCC (P)	4	0.16	CAC (H)	2	0.07	CGC (R)	0	0.00
CUA (L)	5	0.05	CCA (P)	31	1.23	CAA (Q)	29	1.41	CGA (R)	18	1.57
CUG (L)	0	0.00	CCG (P)	0	0.00	CAG (Q)	12	0.59	CGG (R)	4	0.35
AUU (I)	420	1.97	ACU (T)	80	2.67	AAU (N)	242	1.88	AGU (S)	42	1.02
AUC (I)	6	0.03	ACC (T)	1	0.03	AAC (N)	16	0.12	AGC (S)	1	0.02
AUA (M)	301	1.78	ACA (T)	39	1.30	AAA (K)	115	1.76	AGA (S)	59	1.44
AUG (M)	38	0.22	ACG (T)	0	0.00	AAG (K)	16	0.24	AGG (S)	12	0.29
GUU (V)	88	2.26	GCU (A)	51	2.76	GAU (D)	56	1.75	GGU (G)	64	1.56
GUC (V)	0	0.00	GCC (A)	1	0.05	GAC (D)	8	0.25	GGC (G)	3	0.07
GUA (V)	62	1.59	GCA (A)	17	0.92	GAA (E)	53	1.49	GGA (G)	72	1.76
GUG (V)	6	0.15	GCG (A)	5	0.27	GAG (E)	18	0.51	GGG (G)	25	0.61

Note: The symbol “*” represents stop codons in the mitochondrial genome of *Trichogramma vitessoides* sp. nov.

### Transfer RNAs, ribosomal RNAs, and D-loop

**Figure 6.**
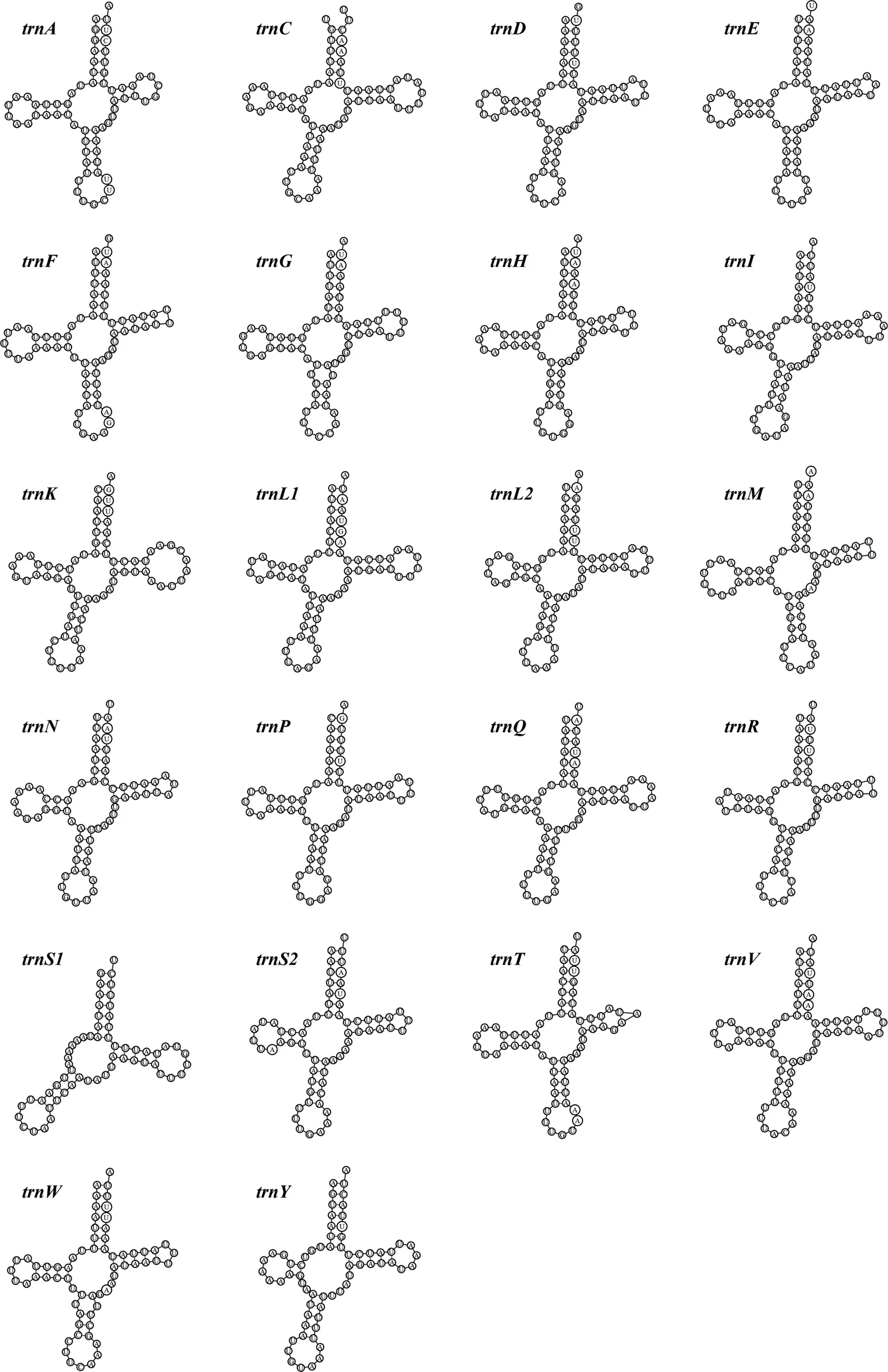
Predicted secondary structures of the 22 mitochondrial tRNAs of *Trichogramma vitessoides* sp. nov.

The 22 tRNA genes range from 60 to 70 bp in length and have a combined length of 1,448 bp, accounting for 8.9% of the mitogenome. Of the 22 tRNA genes, 18 are encoded on the H strand and four (*trnS2*, *trnT*, *trnK*, and *trnE*) on the L strand. Twenty-one tRNAs exhibit the typical cloverleaf secondary structure, whereas *trnS1* lacks a conventional dihydrouridine arm (Fig. 6). The two rRNA genes are encoded on the H strand and are separated by *trnG* and *trnA*. The *rrnS* gene is 760 bp long and the *rrnL* gene is 1,379 bp long; both have an A+T content of 88.3%. The 840-bp D-loop is located between *trnW* and *trnM* and has an A+T content of 88.9% (Tables 5, 6).

### Variation in Ka/Ks ratios among mitochondrial protein-coding genes

**Figure 7.**
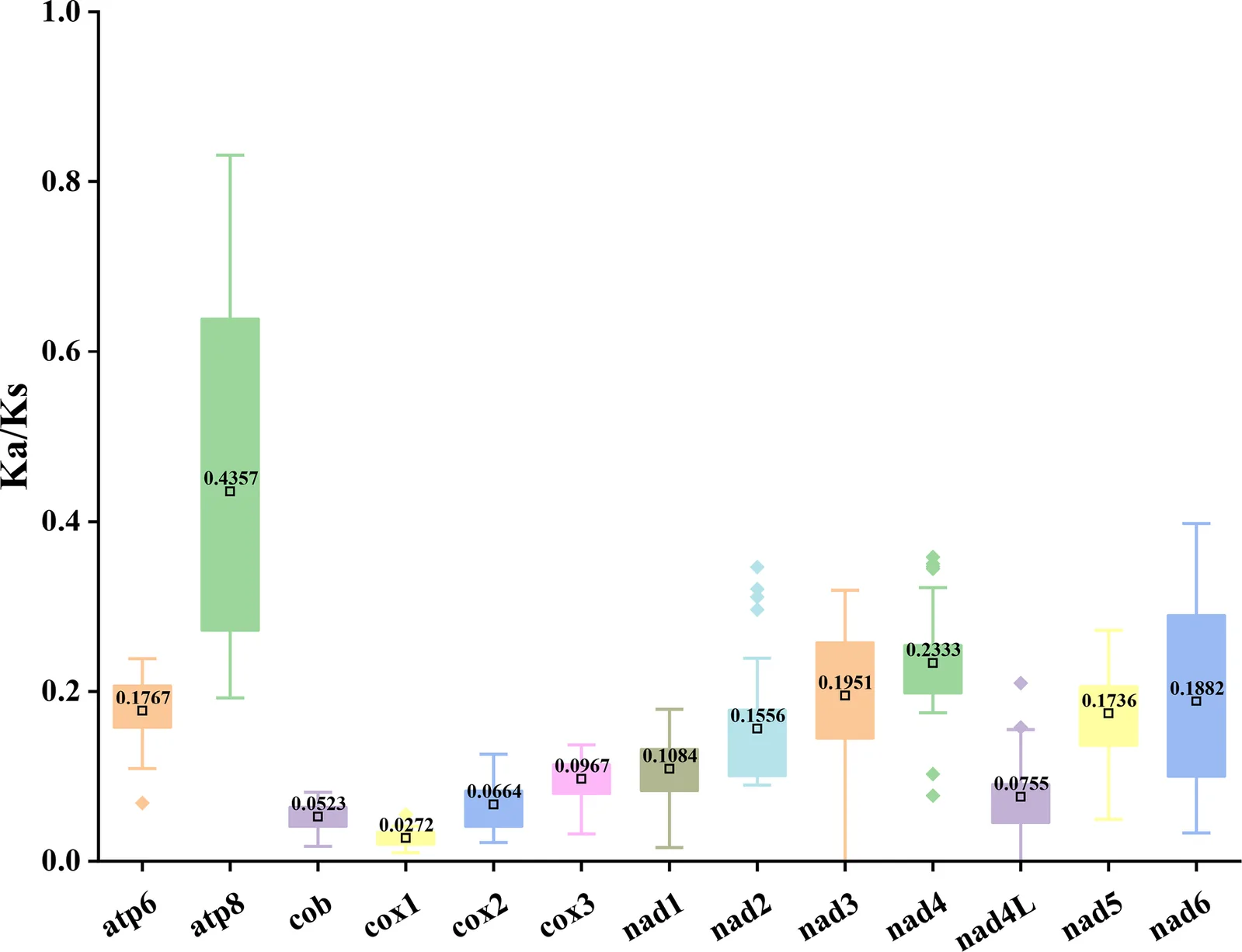
Distribution of *Ka*/*Ks* ratios for the 13 mitochondrial protein-coding genes across the eight sampled *Trichogramma* species. Squares and adjacent values indicate the mean ratio for each gene.

*Ka*/*Ks* ratios were calculated for all pairwise comparisons among the eight sampled *Trichogramma* species and summarised as a mean value for each mitochondrial PCG (Fig. 7). Mean *Ka*/*Ks* ratios increased in the following order: *cox1* < *cob* < *cox2* < *nad4L* < *cox3* < *nad1* < *nad2* < *nad5* < *atp6* < *nad6* < *nad3* < *nad4* < *atp8*. Mean values ranged from 0.0272 for *cox1* to 0.4357 for *atp8*, and all 13 PCGs had mean *Ka*/*Ks* ratios below 1. Among the 13 PCGs, *cox1* showed the lowest mean *Ka*/*Ks* ratio, followed by *cob* and *cox2*, indicating comparatively stronger evolutionary constraint, whereas *atp8* showed the highest mean ratio, followed by *nad4* and *nad3*, suggesting relatively relaxed selective constraints and a greater tolerance of nonsynonymous substitutions. Because the present analysis was conducted at the gene level, it does not identify individual conserved or variable nucleotide or amino-acid sites.

### Phylogenetic analysis

**Figure 8.**
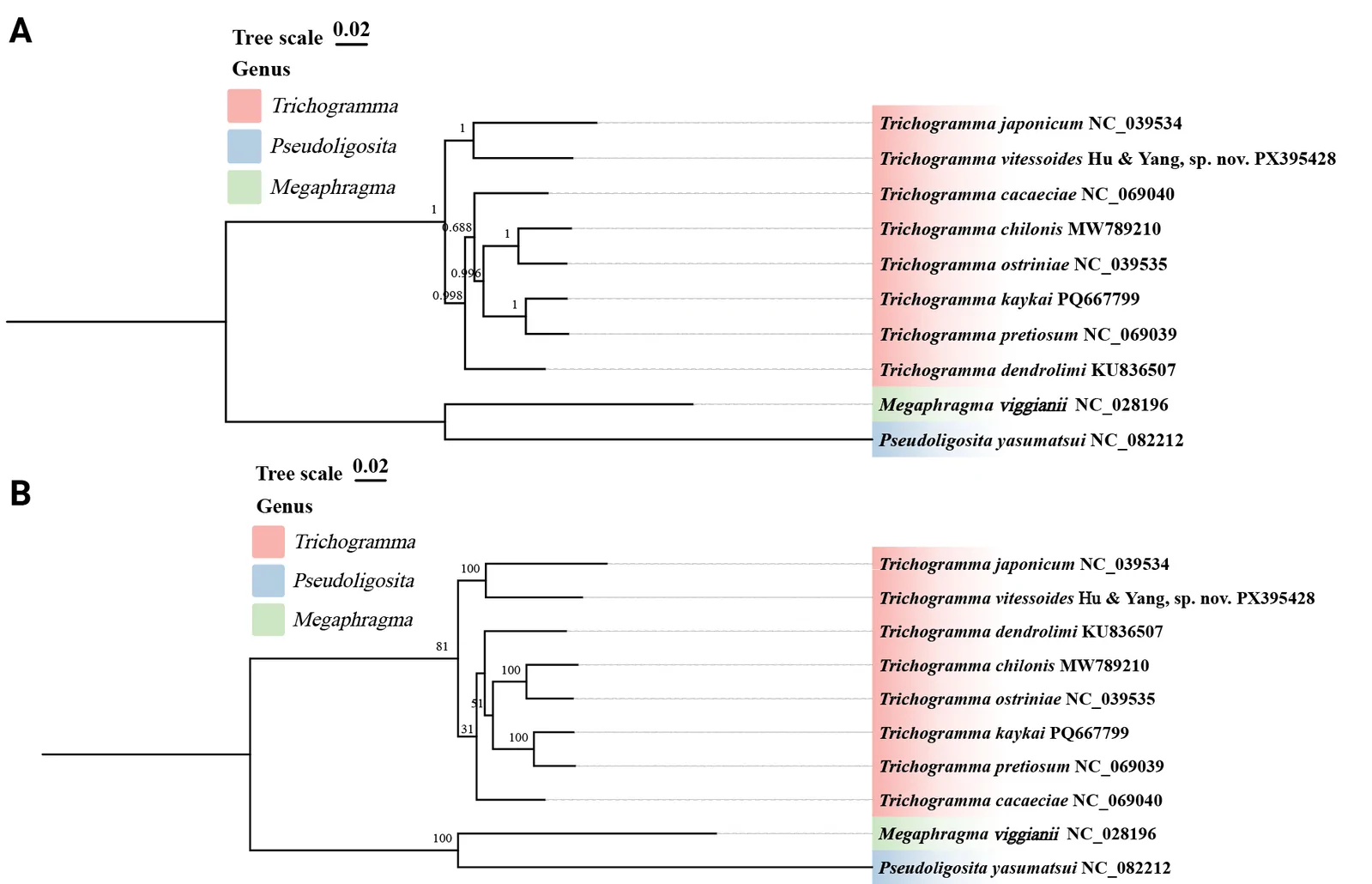
Phylogenetic relationships inferred from the concatenated amino-acid sequences of the 13 mitochondrial protein-coding genes. **A**. Bayesian inference tree; **B**. Maximum-likelihood tree. Numbers adjacent to nodes indicate Bayesian posterior probabilities in A and ultrafast bootstrap support values in B. *Pseudoligosita yasumatsui* and *Megaphragma viggianii* were used as outgroups. GenBank accession numbers are shown after taxon names.

BI and ML analyses of the concatenated amino-acid sequences of the 13 PCGs produced largely congruent topologies (Fig. 8). The two analyses differed only in the relative placement of *Trichogramma dendrolimi* Matsumura, 1926 and *T. cacaeciae*. In both trees, the sampled *Trichogramma* species formed a well-supported clade relative to the two outgroups, *Pseudoligosita yasumatsui* and *Megaphragma viggianii*. *T. vitessoides* sp. nov. was consistently recovered as the sister taxon to *T. japonicum*, with maximal support in both analyses (Bayesian posterior probability = 1.00; ultrafast bootstrap support = 100). These results support the placement of the new species within *Trichogramma* under the present taxon sampling.

## Discussion

### Taxonomic evidence

Male genital morphology provides the primary morphological basis for recognising *T. vitessoides* sp. nov. The combination of straight chelate structures, gonoforceps slightly shorter than or equal to the chelate structures, a weakly developed median ventral projection, long central ridges, and an aedeagus distinctly longer than its apodemes separates the new species from the compared congeners. Among these species, *T. vitessoides* sp. nov. is most similar to *T. raoi*, but the two differ in the relative positions of the apices of the chelate structures and gonoforceps and in the relative lengths of the aedeagus and its apodemes (Pinto 1999; Lin et al. 2022). These differences, together with the quantitative genital measurements, support the morphological diagnosis of the new species.

### DNA barcode evidence

DNA barcode comparisons complement the morphological evidence. The identical *cox1* and 28S rRNA sequences obtained independently from two females indicate that no variation was detected between the two sampled individuals at these loci. The *cox1* sequence showed more than 99% identity to two unidentified *Trichogramma* records, OP443545 and OP443462, associated with material examined by Nakabayashi and Ohshima (2024). These records may represent *T. vitessoides* sp. nov. or a closely related lineage; however, their identity cannot be established without examination of the corresponding voucher specimens. Among named species, *cox1* p-distances from *T. vitessoides* sp. nov. ranged from 7.26% to 8.12%. The lowest distance was observed with *T. kaykai*, whereas mitogenome-based phylogenetic analyses recovered *T. japonicum* as its sister taxon. This difference is not necessarily contradictory because the short *cox1* fragment represents only one portion of the mitochondrial genome and may contain insufficient information to reproduce relationships inferred from all 13 PCGs. The 28S rRNA D2 sequence also showed relatively low identity to available named *Trichogramma* sequences. Together with the diagnostic male genital characters, these molecular differences support the recognition of the sampled lineage as a distinct species. Nevertheless, DNA sequence divergence was treated as supporting evidence rather than as an independent species-delimitation criterion.

### Mitogenome organisation and nucleotide composition

The 16,221-bp mitochondrial genome of *T. vitessoides* sp. nov. contains the typical complement of 37 genes and one D-loop. Previous studies have shown that *Trichogramma* mitogenomes are extensively rearranged relative to the inferred ancestral insect gene order (Chen et al. 2018a; Yan et al. 2022). Within the congeneric taxa sampled here, however, the relative order of the 37 mitochondrial genes was conserved. In particular, *T. vitessoides* sp. nov. shared the same gene order and strand orientation with *T. japonicum* and *Trichogramma ostriniae* Pang & Chen, 1974 (Chen et al. 2018a), whereas strand orientation differed for some genes in other sampled congeners. These results indicate conservation of relative gene order among the sampled taxa, but this pattern should not be generalised to all species of *Trichogramma* without broader taxon sampling. The mitogenome has an overall A+T content of 84.4%, and the 13 PCGs have an A+T content of 82.0%. These values are consistent with the strong A+T bias commonly reported in hymenopteran mitochondrial genomes (Cameron 2014). The negative AT-skew and positive GC-skew reflect higher frequencies of T than A and G than C, respectively. All PCGs begin with ATN codons; 11 terminate with TAA, whereas *nad4L* and *cox3* terminate with TAG. The predominance of A- and U-ending codons is also consistent with the overall nucleotide composition of the genome (Cameron 2014; Chen et al. 2018a).

### Relative selective constraints

Mean *Ka*/*Ks* ratios were below 1 for all 13 PCGs, consistent with purifying selection across the sampled *Trichogramma* species. The lower ratios observed for *cox1*, *cob*, and *cox2* suggest comparatively stronger selective constraints, whereas the higher ratios of *atp8*, *nad4*, and *nad3* indicate relatively relaxed selective constraints. Similar heterogeneity in selective constraint among mitochondrial PCGs has been reported in metazoan mitogenomes (Castellana et al. 2011). However, because the present analysis was conducted at the gene level, the observed pattern should not be interpreted as evidence of site-specific selection or as identifying particular conserved or variable nucleotide or amino-acid sites.

### Phylogenetic position

Phylogenetic analyses of the concatenated amino-acid sequences of the 13 mitochondrial PCGs consistently placed *T. vitessoides* sp. nov. within the sampled *Trichogramma* clade. The BI and ML topologies were largely congruent, differing only in the relative placement of *T. dendrolimi* and *T. cacaeciae*. In both analyses, *T. vitessoides* sp. nov. was recovered as the sister taxon to *T. japonicum* with maximal nodal support. This relationship supports the generic placement of the new species and indicates a close mitochondrial affinity between the two taxa (Chen et al. 2018a; Yan et al. 2022). All eight sampled *Trichogramma* species formed a well-supported clade relative to the two outgroups. However, because only a small proportion of the described diversity of *Trichogramma* was included, the present analysis should not be interpreted as a comprehensive test of monophyly or intrageneric relationships across the genus. The agreement between male genital morphology, nuclear 28S divergence, and mitochondrial evidence provides complementary support for the taxonomic placement and recognition of *T. vitessoides* sp. nov.

## Conclusions

Using morphological examination, DNA barcode comparisons, and complete mitogenome data, we describe *Trichogramma vitessoides* sp. nov., an egg parasitoid originally recovered from *Heortia vitessoides* on *Aquilaria sinensis* in Guangdong, China. Diagnostic characters of the male genitalia, together with divergence in mitochondrial *cox1* and nuclear 28S rRNA, support its recognition as a distinct species. Phylogenetic analyses of the 13 mitochondrial PCGs placed the new species as the sister taxon to *T. japonicum* under the present taxon sampling. The complete mitogenome expands the available genomic resources for comparative and systematic studies of *Trichogramma*. Further research should assess the host range, life-history traits, mass-rearing suitability, non-target effects, and field performance of *T. vitessoides* sp. nov. before its practical application in integrated pest management.

## Supplementary Material

XML Treatment for Trichogramma vitessoides

## Additional information

### Artificial Intelligence (AI) use

The authors accept full responsibility for the content of the manuscript, including the disclosure of any use of AI.

No AI tools were used in the preparation of this manuscript.

### Author ORCIDs

Z. Ge https://orcid.org/0009-0006-4445-2369

### Data availability

All nucleotide sequences generated in this study have been deposited in GenBank. The complete mitochondrial genome is available under accession number PX395428; the partial mitochondrial cox1 sequences are available under accession numbers PZ626304 and PZ626305; and the nuclear 28S rRNA D2 sequences are available under accession numbers PZ630113 and PZ630114.
